# Trends in Self-reported and Biochemically Verified Cocaine and Methamphetamine Use Among Pregnant Individuals in Northern California, 2011-2019

**DOI:** 10.1001/jamanetworkopen.2022.48055

**Published:** 2022-12-21

**Authors:** Kelly C. Young-Wolff, Natalie E. Slama, Varada Sarovar, Mishka Terplan, Deborah Ansley, Sara R. Adams, Stacey E. Alexeeff

**Affiliations:** 1 Division of Research, Kaiser Permanente Northern California, Oakland; 2 Department of Psychiatry and Behavioral Sciences, University of California, San Francisco; 3 Friends Research Institute, Baltimore, Maryland; 4 Regional Offices, Kaiser Permanente Northern California, Oakland

## Abstract

This cross-sectional study uses data from the Kaiser Permanente Northern California health care system with universal screening via self-report and urine toxicology at prenatal care entrance to examine trends in cocaine and methamphetamine use among pregnant individuals from 2011 to 2019.

## Introduction

Cocaine use and methamphetamine use during pregnancy have potential short- and long-term health consequences.^1,2^ Rates of self-reported prenatal stimulant use have increased globally,^1^ but epidemiologic studies have not included biochemical verification. We examined trends in cocaine and methamphetamine use at prenatal care entrance from 2011 to 2019 using data from the Kaiser Permanente Northern California (KPNC) health care system with universal screening via self-report and urine toxicology.

## Methods

In this cross-sectional study using time-series analysis, pregnant individuals universally screened for cocaine and methamphetamine use by (1) self-report, defined as any “use since pregnancy,” and (2) urine toxicology testing at entrance to prenatal care (median gestation, 8 [IQR, 7-10] weeks) in KPNC from January 1, 2011, to December 31, 2019, were considered for inclusion. For individuals with multiple pregnancies, all pregnancies meeting eligibility criteria were included. The KPNC institutional review board approved this study and waived consent because research involved no more than minimal risk to participants and could not practicably be performed without a waiver. This study followed the STROBE reporting guideline.

Statistical analysis was conducted from August 9 to September 8, 2022. We separately estimated the adjusted prevalence of cocaine and methamphetamine use via self-report and/or toxicology annually using Poisson regression with a log-link function, accounting for overdispersion, using SAS, version 9.4 (SAS Institute Inc). Prevalence estimates were adjusted for age, race and ethnicity (reported by patients supplemented with administrative records), and census-tract neighborhood deprivation index (NDI) using mean covariate distributions. Linear trends were modeled with a calendar year term. We also estimated the relative rate for each year compared with the previous year (eg, 2012 vs 2011). All *P* values were from 2-sided tests, and results were deemed statistically significant at *P* < .05.

## Results

Of 364 284 eligible pregnancies, 38 233 without toxicology test results were excluded. The sample (N = 326 051) was 26.2% Asian or Pacific Islander, 6.3% Black, 25.8% Hispanic, 37.1% non-Hispanic White, and 4.6% other, multiracial, or unknown (the “other” category includes Native Americans; multiracial includes any person with ≥1 of the following reported categories: Asian or Pacific Islander, Black, White, or Native American). A total of 0.9% the sample were aged 12 to 17 years, 14.1% were aged 18 to 24 years, 62.8% were aged 25 to 34 years, and 22.2% were older than 34 years; the median NDI was –0.272 (IQR, –0.733 to 0.393).

From 2011 to 2019, the adjusted prevalence of any prenatal cocaine use increased from 0.10% (95% CI, 0.07%-0.13%) to 0.15% (95% CI, 0.12%-0.18%) at an annual relative rate of 1.09 (95% CI, 1.05-1.12) (Figure, A; Table). Annual relative rate increases for cocaine use were estimated to be greater by toxicology tests (1.13, 95% CI, 1.09-1.18) than by self-report (1.06, 95% CI, 1.02-1.10).

**Figure.**
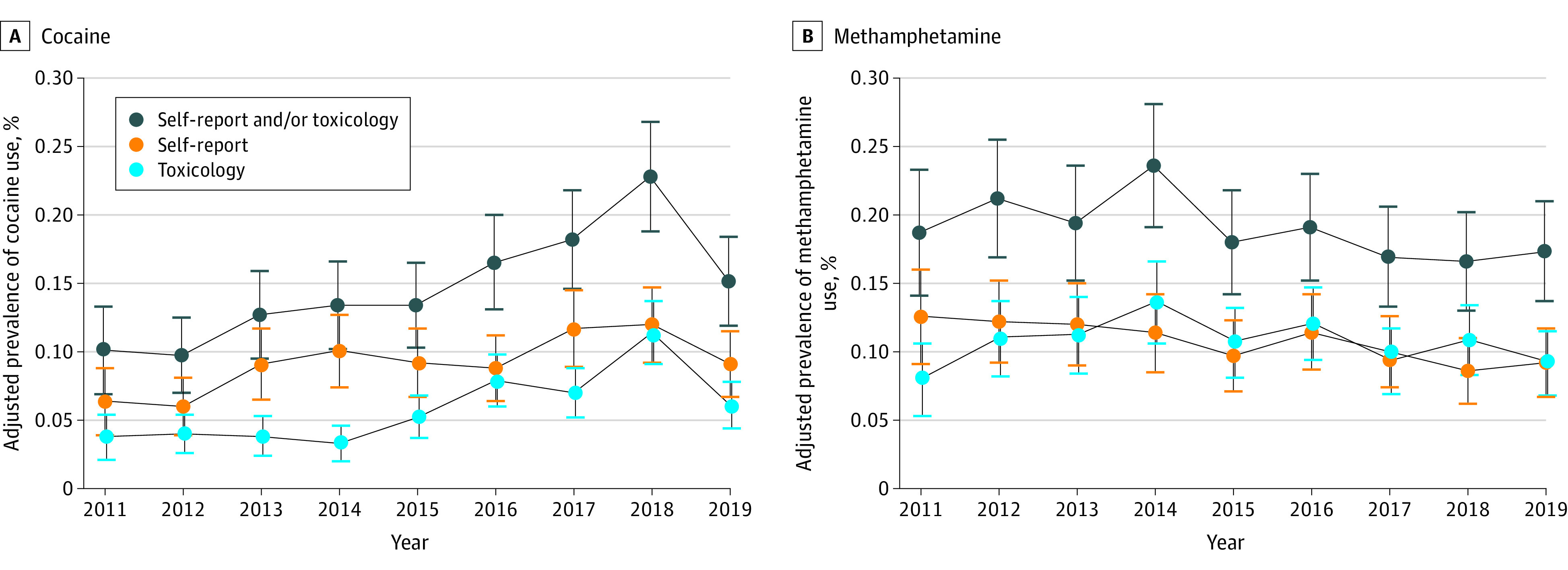
Adjusted Prevalence of Prenatal Cocaine and Methamphetamine Use in Kaiser Permanente Northern California (KPNC), Overall and by Screening Type, 2011-2019 (326 051 Pregnancies) Error bars indicate 95% CIs of adjusted prevalences. Cocaine screening tests were performed on a Beckman Coulter AU680 chemistry analyzer using the Emit II Plus Cocaine Metabolite Assay (Siemens Medical Solutions USA Inc), which detects the cocaine metabolite benzoylecgonine with a positive cutoff of 150 ng/mL (to convert to nanomoles per liter, multiply by 3.297). Confirmatory testing for the presence of benzoylecgonine was performed by liquid chromatography–tandem mass spectrometry (LC-MS/MS) for all positive immunoassay results. The positive cutoff for the confirmation assay was 100 ng/mL. Among pregnant individuals who screened positive for any prenatal cocaine use, 59.8% were positive on self-report only, 37.9% were positive on toxicology only, and 2.3% were positive on both self-report and toxicology. Amphetamine screening tests were performed on a Beckman Coulter AU680 chemistry analyzer using the DRI Amphetamines Assay (Thermo Fisher Scientific) with a positive cutoff of 500 ng/mL (to convert to nanomoles per liter, multiply by 7.4). This immunoassay does not distinguish between amphetamine and methamphetamine, and any positive test is reported as “T’FOLLOW” with confirmatory testing to distinguish between amphetamine species. Confirmatory testing for the presence of methamphetamine was performed by LC-MS/MS for all positive immunoassay results, with a positive cutoff of 250 ng/mL. Among pregnant individuals who screened positive for any prenatal methamphetamine use, 43.4% were positive on self-report only, 43.6% were positive on toxicology only, and 13.0% were positive on both self-report and toxicology. The sample of individuals included in the study was generally representative of the population of all pregnant individuals receiving care from KPNC. There were only small differences in the demographic characteristics of individuals who were included vs excluded (due to missing data on urine toxicology tests), indicating minimal to no selection bias.

**Table.** Adjusted Prevalence of Stimulant Use During Early Pregnancy in KPNC for Each Year (2011-2019) and Annual Relative Rate of Change, by Stimulant Use (N = 326 051)

Stimulate use	Adjusted prevalence of stimulant use during pregnancy, % (95% CI)^a^									Linear trend estimation	
	2011	2012	2013	2014	2015	2016	2017	2018	2019	Annual relative rate of change estimate (95% CI)	*P* value
**Cocaine use**											
Self-report or toxicology	0.10 (0.07-0.13)	0.10 (0.07-0.13)	0.13 (0.09-0.16)	0.13 (0.10-0.17)	0.13 (0.10-0.17)	0.17 (0.13-0.20)	0.18 (0.15-0.22)	0.23 (0.19-0.27)	0.15 (0.12-0.18)	NA	NA
Annual relative rate	NA	0.96 (0.63-1.48)	1.30 (0.89-1.90)	1.06 (0.75-1.50)	1.00 (0.72-1.40)	1.24 (0.90-1.69)	1.10 (0.82-1.46)	1.25 (0.96-1.63)	0.66 (0.50-0.88)	1.09 (1.05-1.12)	<.001
Toxicology	0.04 (0.02-0.05)	0.04 (0.03-0.05)	0.04 (0.02-0.05)	0.03 (0.02-0.05)	0.05 (0.04-0.07)	0.08 (0.06-0.10)	0.07 (0.05-0.09)	0.11 (0.09-0.14)	0.06 (0.04-0.08)	NA	NA
Annual relative rate	NA	1.06 (0.61-1.86)	0.96 (0.57-1.61)	0.87 (0.51-1.50)	1.57 (0.96-2.57)	1.51 (1.02-2.22)	0.88 (0.62-1.26)	1.63 (1.17-2.26)	0.54 (0.38-0.76)	1.13 (1.09-1.18)	<.001
Self-report	0.06 (0.04-0.09)	0.06 (0.04-0.08)	0.09 (0.07-0.12)	0.10 (0.07-0.13)	0.09 (0.07-0.12)	0.09 (0.06-0.11)	0.12 (0.09-0.14)	0.12 (0.09-0.15)	0.09 (0.07-0.11)	NA	NA
Annual relative rate	NA	0.95 (0.57-1.59)	1.52 (0.97-2.37)	1.10 (0.75-1.62)	0.91 (0.63-1.33)	0.96 (0.66-1.41)	1.32 (0.92-1.89)	1.02 (0.84-1.18)	0.93 (0.78-1.10)	1.06 (1.02-1.10)	.002
**Methamphetamine use**											
Self-report or toxicology	0.19 (0.14-0.23)	0.21 (0.17-0.25)	0.19 (0.15-0.24)	0.24 (0.19-0.28)	0.18 (0.14-0.22)	0.19 (0.15-0.23)	0.17 (0.13-0.21)	0.17 (0.13-0.20)	0.17 (0.14-0.21)	NA	NA
Annual relative rate	NA	1.13 (0.82-1.56)	0.92 (0.68-1.23)	1.22 (0.91-1.62)	0.76 (0.57-1.01)	1.06 (0.79-1.42)	0.89 (0.66-1.19)	0.98 (0.72-1.33)	1.04 (0.77-1.41)	0.97 (0.95-1.00)	.05
Toxicology	0.08 (0.05-0.11)	0.11 (0.08-0.14)	0.11 (0.08-0.14)	0.14 (0.11-0.17)	0.11 (0.08-0.13)	0.12 (0.09-0.15)	0.09 (0.07-0.12)	0.11 (0.08-0.13)	0.09 (0.07-0.12)	NA	NA
Annual relative rate	NA	1.38 (0.91-2.09)	1.02 (0.72-1.45)	1.22 (0.87-1.69)	0.79 (0.57-1.09)	1.13 (0.81-1.57)	0.77 (0.55-1.09)	1.16 (0.82-1.64)	0.85 (0.60-1.20)	0.99 (0.96-1.03)	.68
Self-report	0.13 (0.09-0.16)	0.12 (0.09-0.15)	0.12 (0.09-0.15)	0.11 (0.09-0.14)	0.10 (0.07-0.12)	0.11 (0.09-0.14)	0.10 (0.07-0.13)	0.09 (0.06-0.11)	0.09 (0.07-0.12)	NA	NA
Annual relative rate	NA	0.97 (0.67-1.40)	0.98 (0.69-1.40)	0.95 (0.67-1.35)	0.85 (0.59-1.23)	1.18 (0.82-1.69)	0.87 (0.61-1.25)	0.86 (0.59-1.26)	1.08 (0.73-1.58)	0.96 (0.93-0.99)	.01

Abbreviations: KPNC, Kaiser Permanente Northern California; NA, not applicable.

^a^ Adjusted prevalence estimates and 95% CIs were estimated from Poisson regression models controlling for age group, race and ethnicity, and neighborhood deprivation index (extracted from the electronic health record).

From 2011 to 2019, the adjusted prevalence of any prenatal methamphetamine use changed from 0.19% (95% CI, 0.14%-0.23%) to 0.17% (95% CI, 0.14%-0.21%) at an annual relative rate of 0.97 (95% CI, 0.95-1.00) (Figure, B; Table). Annual relative rates did not change for methamphetamine use measured by toxicology tests (0.99 [95% CI, 0.96-1.03]) and decreased when measured by self-report (0.96 [95% CI, 0.93-0.99]).

## Discussion

This study found that, consistent with population-level increases in the prevalence of cocaine and methamphetamine use in the general US population,^3^ rates of cocaine use increased modestly among pregnant individuals in KPNC. Although the adjusted prevalence was higher across years by self-report vs toxicology tests, likely reflecting screening for any use since pregnancy vs current use, rates increased faster via toxicology tests. In contrast, rates of prenatal methamphetamine use decreased slightly over time via self-report and did not change via toxicology tests. Although prenatal methamphetamine use was nearly twice as prevalent as prenatal cocaine use in 2011, prevalence rates were similar by 2019. Across years, the adjusted prevalence of cocaine and methamphetamine use was roughly twice as high when including data from self-report and toxicology tests vs either measure on its own, indicating that the combination of both methods may lead to the best identification.

This study was limited to insured patients in Northern California screened for stimulant use during pregnancy. Results do not capture the amount or frequency of use, continued prenatal use, or stimulant use disorders. We are not able to differentiate prenatal use that occurred before vs after pregnancy recognition. Stimulants are detectable via urine 2 to 4 days after a single use but can be detectable longer with long-term use, and some individuals may be misclassified as nonusers. Results may not be generalizable to other states or individuals without health care.

Rates of cocaine use during early pregnancy increased modestly from 2011 to 2019 and likely reflect changes in continuation of prepregnancy use into pregnancy. Differing trends in prenatal cocaine and methamphetamine use highlight the importance of early screening for different types of prenatal illicit stimulant use, assessment of substance use disorder, and linkage to nonpunitive treatment, as needed. Research indicates that stopping stimulant use during pregnancy improves birth outcomes,^4^ and continued research is needed to understand factors associated with different types of prenatal stimulant use over time.

## Notes

Supplement.
Data Sharing Statement
