# Supplementary material for: Trends in Self-reported and Biochemically Verified Cocaine and Methamphetamine Use Among Pregnant Individuals in Northern California, 2011-2019
Source: JAMA Netw Open. 2022 Dec 21;5(12):e2248055. doi: 10.1001/jamanetworkopen.2022.48055 (PMC9857285; doi:10.1001/jamanetworkopen.2022.48055)
Supplement: Supplement. — Data Sharing Statement [file jamanetwopen-e2248055-s001.pdf]

Data Sharing Statement

Young-Wolff. Trends in Self-reported and Biochemically Verified Cocaine and Methamphetamine Use Among Pregnant Individuals in Northern California, 2011-2019. *JAMA Netw Open*. Published December 21, 2022. doi:10.1001/jamanetworkopen.2022.48055

Data
Data available: No
